# Origin of the spin Seebeck effect in compensated ferrimagnets

**DOI:** 10.1038/ncomms10452

**Published:** 2016-02-04

**Authors:** Stephan Geprägs, Andreas Kehlberger, Francesco Della Coletta, Zhiyong Qiu, Er-Jia Guo, Tomek Schulz, Christian Mix, Sibylle Meyer, Akashdeep Kamra, Matthias Althammer, Hans Huebl, Gerhard Jakob, Yuichi Ohnuma, Hiroto Adachi, Joseph Barker, Sadamichi Maekawa, Gerrit E. W. Bauer, Eiji Saitoh, Rudolf Gross, Sebastian T. B. Goennenwein, Mathias Kläui

**Affiliations:** 1 Walther-Meißner-Institut, Bayerische Akademie der Wissenschaften, 85748 Garching, Germany; 2 Institute of Physics, Johannes Gutenberg-University Mainz, 55099 Mainz, Germany; 3 Materials Science in Mainz, Staudinger Weg 9, 55128 Mainz, Germany; 4 Physik-Department, Technische Universität München, 85748 Garching, Germany; 5 WPI Advanced Institute for Materials Research, Tohoku University, Sendai 980-8577, Japan; 6 ERATO, Japan Science and Technology Agency, Sendai 980-8577, Japan; 7 Kavli Institute of NanoScience, Delft University of Technology, 2628 Delft, The Netherlands; 8 Nanosystems Initiative Munich (NIM), Schellingstraße 4, 80799 München, Germany; 9 Institute for Materials Research, Tohoku University, Sendai 980-8577, Japan; 10 Advanced Science Research Center, Japan Atomic Energy Agency, Tokai 319-1195, Japan; 11 CREST, Japan Science and Technology Agency, Tokyo 102-0076, Japan

## Abstract

Magnons are the elementary excitations of a magnetically ordered system. In ferromagnets, only a single band of low-energy magnons needs to be considered, but in ferrimagnets the situation is more complex owing to different magnetic sublattices involved. In this case, low lying optical modes exist that can affect the dynamical response. Here we show that the spin Seebeck effect (SSE) is sensitive to the complexities of the magnon spectrum. The SSE is caused by thermally excited spin dynamics that are converted to a voltage by the inverse spin Hall effect at the interface to a heavy metal contact. By investigating the temperature dependence of the SSE in the ferrimagnet gadolinium iron garnet, with a magnetic compensation point near room temperature, we demonstrate that higher-energy exchange magnons play a key role in the SSE.

## Abstract (web summary)

Ferrimagnets possess multiple spin sub-lattices resulting in a complex magnon band structure and subtle spin transport across interfaces. Here, the authors show how the spin Seebeck effect, the thermal generation of pure spin current, may be an effective tool to study these magnetic excitations.

## Graphical abstract

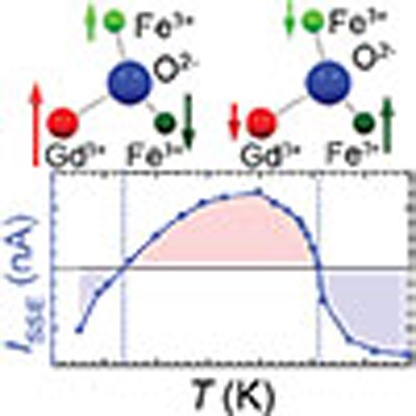

Recently, the spin Seebeck effect (SSE)^1^,^2^,^3^,^4^ has attracted much attention, due to open fundamental physics questions^1^,^2^,^3^,^5^,^6^,^7^,^8^,^9^,^10^,^11^,^12^,^13^, and also because of the potential for practical applications^14^. It is now widely accepted that the thermopower signals measured in ferromagnetic insulator/normal metal (FMI/NM) bilayers in the longitudinal SSE geometry are indeed a consequence of magnonic spin currents generated by a temperature gradient^2^,^5^,^6^,^7^,^8^,^15^. However, the exact mechanism of the SSE and the contribution of different magnon modes to the spin current are still under discussion^3^,^9^,^10^,^11^,^12^,^13^. So far, the detection of the magnonic spin currents via the inverse spin Hall effect (ISHE) in the adjacent normal metal layer has been analysed based on an effective spin current combined with an effective spin mixing conductance that governs the spin current transport across the interface. Experimental data have typically been described within a simple spin wave model, neglecting higher energy magnon bands^3^,^15^. This approach is reasonable for the SSE in ferromagnets/normal metal hybrids, where one only has to consider low-energy magnons. However, owing to the presence of different magnetic sublattices, ferrimagnets exhibit more complex magnon spectra, much like acoustic and optical phonon modes in crystals with more than one atom per unit cell^16^,^17^. For Gd_3_Fe_5_O_12_/Pt (GdIG/Pt) bilayers as a test bed, we demonstrate that the SSE in complex ferrimagnets results from the balance of thermal spin pumping between multiple magnon modes where distinct magnetic moments might contribute differently to the spin mixing conductance. This leads to different efficiencies for the spin transport across the GdIG/Pt interface. In particular, we measure the magneto-thermopower (the longitudinal SSE) as a function of temperature. We observe an abrupt sign change in the SSE close to the magnetic compensation point, followed by another, more gradual sign change at lower temperatures demonstrating that the SSE is qualitatively different to the temperature dependence of the net magnetization. Our theoretical analysis reveals that the thermally generated net spin current giving the SSE signal reflects the complex interplay of two magnon branches and possibly the exchange coupling at the interface. This shows that the description of the SSE needs to take into account the magnon dispersion relation including the polarization of spin waves as well as interface effects that have previously been neglected.

## Results

### Magnetic properties of GdIG thin films

**Figure 1:**
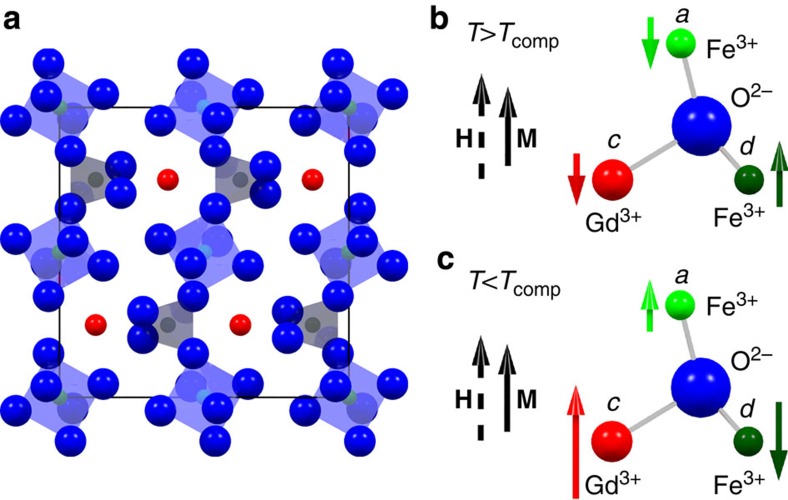
Magnetic sublattices of GdIG. (**a**) Iron garnet crystal structure. The tetrahedrally and octahedrally coordinated Fe^3+^ ions are highlighted. (**b**,**c**) The three different magnetic sublattices of GdIG shown for temperatures above (*T*>*T*_comp_) and below (*T*<*T*_comp_) the magnetic compensation temperature *T*_comp_. Due to the strong temperature dependence of the magnetic Gd sublattice (*c* sites), the Gd sublattice dominates the magnetic behaviour for *T*<*T*_comp_, while for *T*>*T*_comp_ it is mainly caused by the antiferromagnetically coupled Fe sublattices (*d* and *a* sites). In the presence of a finite magnetic field, the net magnetization **M** points along the external magnetic field **H**. The direction and length of the coloured arrows represent the direction and magnitude of the magnetizations of the three magnetic sublattices.

First, we discuss the magnetic properties of GdIG as a function of temperature. As sketched in Fig. 1, GdIG is a ferrimagnetic insulator with three magnetic sublattices. Each unit cell consists of 12 trivalent Fe atoms that are tetrahedrally coordinated with oxygen atoms (*d* sites), 8 Fe atoms that are octahedrally coordinated (*a* sites) and 12 dodecahedrally coordinated Gd atoms (*c* sites). The two Fe sublattices are strongly coupled via antiferromagnetic superexchange (exchange constant *J*_ad_≃32 cm^−1^)^16^, with a Néel temperature of *T*_N_≃550 K. Since the Gd moments are weakly exchange coupled to the Fe *a* sublattices (*J*_ac_≃7 cm^−1^)^16^ they experience an effective magnetic field below *T*_N_ and can be treated as an ‘exchange-enhanced' paramagnetic moment^18^, which is strongly temperature dependent. The magnetization of GdIG is thus determined by the *d* site Fe ions at high temperatures (Fig. 1b). With decreasing temperature, the magnetization of the Gd sublattice strongly increases, and together with the Fe magnetization at the *a* site eventually overcomes the Fe magnetization at the *d* site. At the magnetic compensation temperature *T*_comp_≃288 K (bulk value)^19^,^20^, the total magnetization of the *a* (Fe) and *c* (Gd) sublattices is equal in magnitude but antiparallel to the magnetization of the *d* (Fe) sublattice, such that the net remanent magnetization of GdIG vanishes. In a finite external magnetic field the net GdIG magnetization exhibits a minimum at *T*_comp_, caused by a reorientation of the sublattice magnetizations (Fig. 1c).

**Figure 2:**
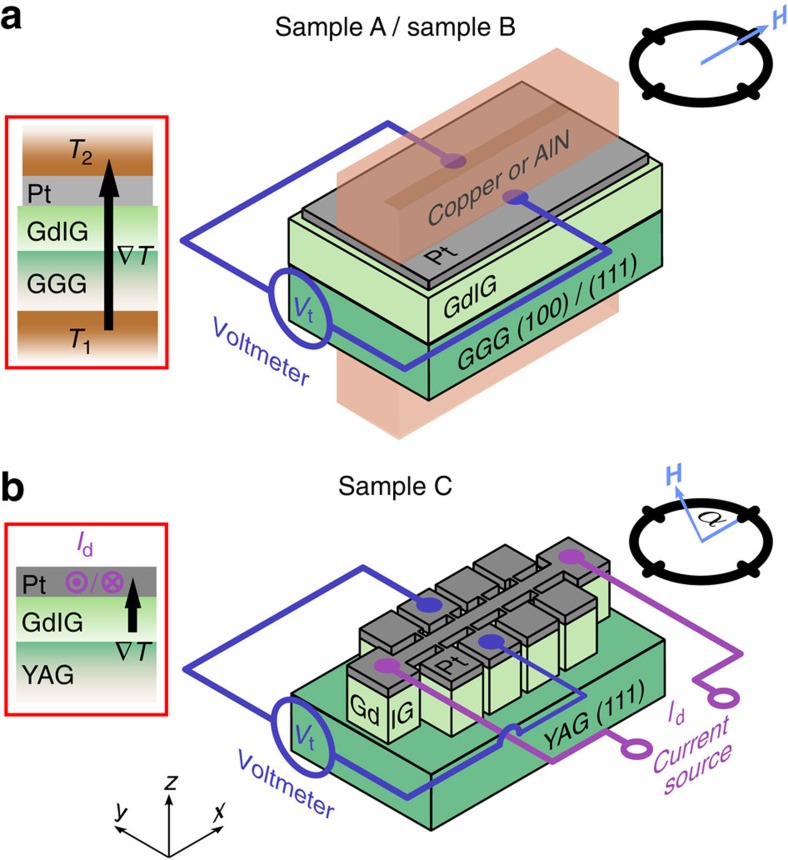
Experimental set-up. (**a**,**b**) Sketches of the experimental configurations used to record the longitudinal spin Seebeck effect (SSE). Three different samples were investigated: Sample A and sample B consist of a GdIG(100 nm)/Pt(8 nm) and a GdIG(1 μm)/Pt(10 nm) bilayer fabricated on a (100)- and a (111)-oriented gadolinium gallium garnet (Gd_3_Ga_5_O_12_, GGG) substrate, respectively. Sample C is composed of a GdIG(26 nm)/Pt(10 nm) bilayer fabricated on a (111)-oriented yttrium aluminium garnet (Y_3_Al_5_O_12_, YAG) substrate. For sample A and B, the longitudinal SSE signal is determined by measuring the transverse voltage *V*_t_ perpendicular to an external magnetic field while modifying the magnetic field magnitude at a fixed magnetic field orientation 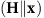. The temperature gradient required for SSE measurements is generated by two independently heated copper blocks or AlN ceramics, respectively. The longitudinal SSE signal of sample C is obtained by recording *V*_t_ as a function of the in-plane orientation of the external magnetic field *α* at a fixed magnetic field magnitude of 2 T. Here the temperature gradient across the GdIG/Pt interface is generated by driving a large current *I*_d_ along the Pt microstructure. The temperature-dependent resistance of the Pt is exploited for on-chip thermometry.

**Figure 3:**
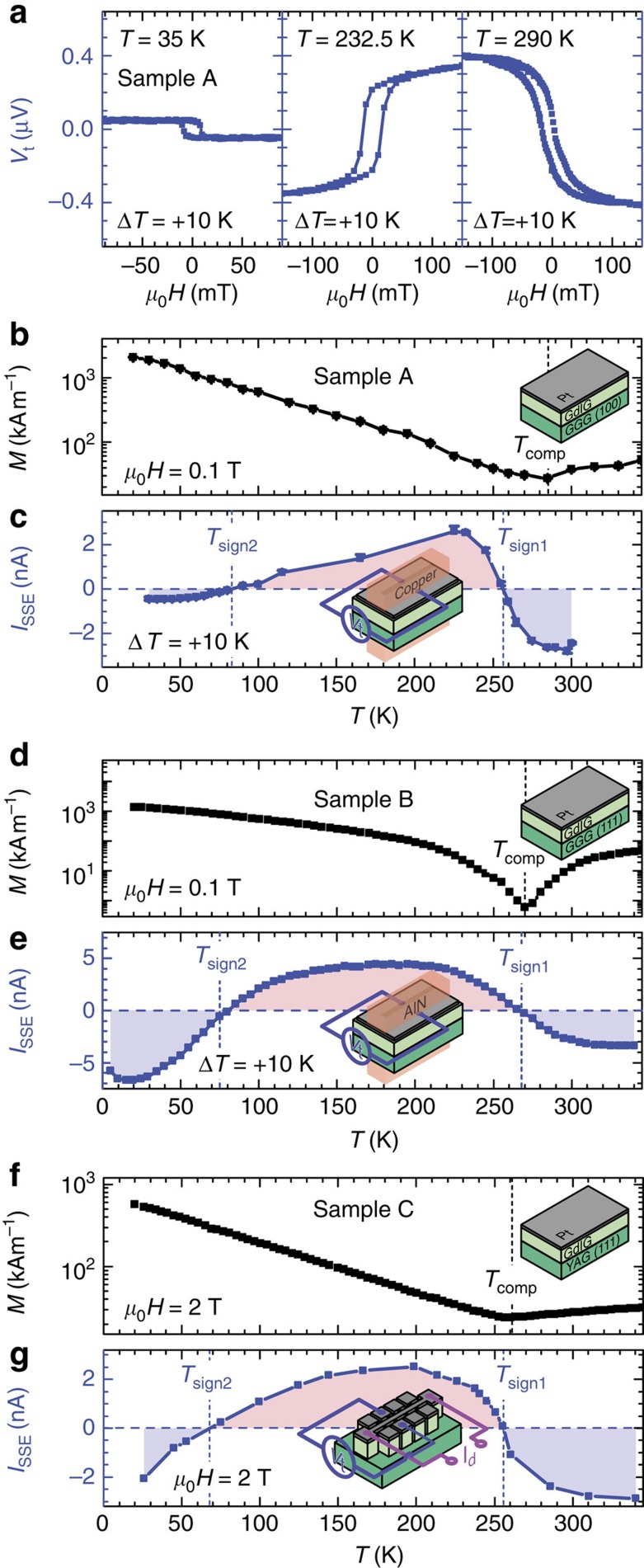
Magnetization and spin Seebeck effect in GdIG/Pt hybrids. (**a**) Transverse voltage *V*_t_ of sample A plotted versus the applied magnetic field for selected temperatures. The hysteresis loop flips sign twice with decreasing temperature. (**b**) Temperature-dependent magnetization of sample A recorded at a magnetic field of 0.1 T. (**c**) Corresponding SSE signal *I*_SSE_ obtained from the difference in *V*_t_ at positive and negative saturation taking the temperature dependence of the Pt resistance *R*(*T*) into account. (**d**,**f**) Magnetization as a function of temperature of sample B and sample C measured at *μ*_0_*H*=0.1 T and *μ*_0_*H*=2 T, respectively. (**e**) *I*_SSE_ signal of sample B. (**g**) *I*_SSE_ signal of sample C obtained by recording the transverse voltage *V*_t_ as a function of the in-plane orientation of the external magnetic field with constant magnitude of 2 T while applying a heating current *I*_d_ of 6 mA across the Hall bar. The SSE signal *I*_SSE_ is then calculated from 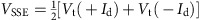. The blue dashed lines mark the zero-crossing temperatures *T*_sign1_ and *T*_sign2_ of the *I*_SSE_ signal. The temperatures *T*_comp_ of the magnetic compensation points are indicated by the black dashed lines.

We investigate the SSE in three samples with different crystallographic orientations and thicknesses (sample A, sample B, and sample C) (Fig. 2). As evident from Fig. 3b,d,f, the magnetic properties of these GdIG thin films recorded by SQUID magnetometry show the characteristics expected for a ferrimagnet with a magnetization compensation point^21^. The magnetization first decreases with decreasing temperature, reaches a minimum at *T*_comp_ and then increases again. The compensation temperature of sample A is around 285 K (Fig. 3b), in line with literature^19^,^20^. For sample B and sample C, *T*_comp_ is somewhat lower (Fig. 3d,f), most likely due to a small reduction of the magnetic exchange strength due to strain effects, Fe deficiency or interdiffusion of Al in GdIG thin films^22^.

### Temperature-dependent spin Seebeck experiments

We now turn to the results of the SSE experiments shown in Fig. 3. The longitudinal SSE was recorded as a function of temperature using two complementary methods. Sample A and sample B are sandwiched between two copper blocks and two AlN ceramics, respectively (Fig. 2a)^6^. A resistive heater as well as a temperature sensor within each block allow the temperature to be independently set and stabilized. To determine the longitudinal SSE signal, the transverse voltage *V*_t_ perpendicular to an external magnetic field is measured while modifying the magnetic field magnitude at a fixed magnetic field orientation 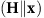. The GdIG/Pt bilayer of sample C is micropatterned into a Hall bar structure using optical lithography and Argon ion beam milling (Fig. 2b). The temperature gradient across the GdIG/Pt interface required for SSE experiments is generated by driving a large current *I*_d_ along the Pt microstructure itself, while simultaneously exploiting the temperature-dependent resistance of the Pt for on-chip thermometry^23^. Here *V*_t_ is recorded as a function of the in-plane external magnetic field direction *α* at a fixed magnetic field magnitude of 2 T. The SSE data shown in Fig. 3a were taken on sample A at three different base temperatures with a temperature difference of Δ*T*=+10 K applied across the sample. Positive temperature difference hereby means that the Pt is warmer than the GdIG and the GGG substrate. As expected for the SSE, a hysteretic voltage signal *V*_t_ is obtained on sweeping the magnetic field^2^. Interestingly, however, the *V*_t_ hysteresis loop flips sign twice with decreasing temperature: while *V*_t_(*H*) is ‘regular' (that is, the *V*_t_(*H*) recorded in GdIG/Pt hybrids has the same sign as the established SSE signal of YIG/Pt; ref. 24) for *T*=280 K, the *V*_t_(*H*) loop recorded for *T*=232.5 K is ‘inverted', while the loop at *T*=35 K is ‘regular' again.

To analyse the temperature-dependent evolution of the SSE signal in more detail, we define the SSE amplitude *V*_SSE_ as the difference 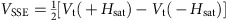, recorded at external magnetic field strengths *μ*_0_*H*_sat_ large enough to saturate the GdIG magnetization. Taking the temperature dependence of the Pt resistance *R*(*T*) into account, Fig. 3c shows the *I*_SSE_(*T*)=*V*_SSE_(*T*)/*R*(*T*) curve from sample A. At first, the SSE signal is negative at high temperatures, then becomes positive in a temperature interval *T*_sign2_≲*T*≲*T*_sign1_ with *T*_sign2_≈80 K, and is negative again for *T*≲*T*_sign2_. The first sign change around *T*_sign1_≈256.5 K is rather abrupt, while the second around *T*_sign2_ is much more gradual. As evident from Fig. 3e,g the SSE signal *I*_SSE_ of sample B and sample C confirm the SSE temperature dependence observed in sample A. Interestingly, the temperature dependence of the SSE is very similar to the Faraday response of GdIG single crystals^25^, demonstrating that the measured SSE is dominated by GdIG bulk properties. However, interface effects might also have an influence on the SSE signal. For example, a layer with modified magnetic properties might form at the interface due to reduced exchange interaction strength at the GdIG surface. However, so far, there is no experimental evidence for the existence of such a magnetic surface layer in other temperature-dependent measurements of the SSE^26^,^27^.

### Theory of SSE in compensated ferrimagnets

To understand and explain the temperature dependence of the SSE signal in GdIG/Pt hybrids in more detail, we carried out theoretical calculations. Using two approaches, one based on a detailed atomistic model of GdIG and the other on a two sublattice linear response formalism, two possible effects can be identified contributing to the observed behaviour: (a) the temperature dependence of the different branches of the magnon dispersion and (b) different spin transport efficiencies across the GdIG/Pt interface, owing to a difference in the exchange coupling strength between the GdIG constituent ions and the Pt conduction electrons.

**Figure 4:**
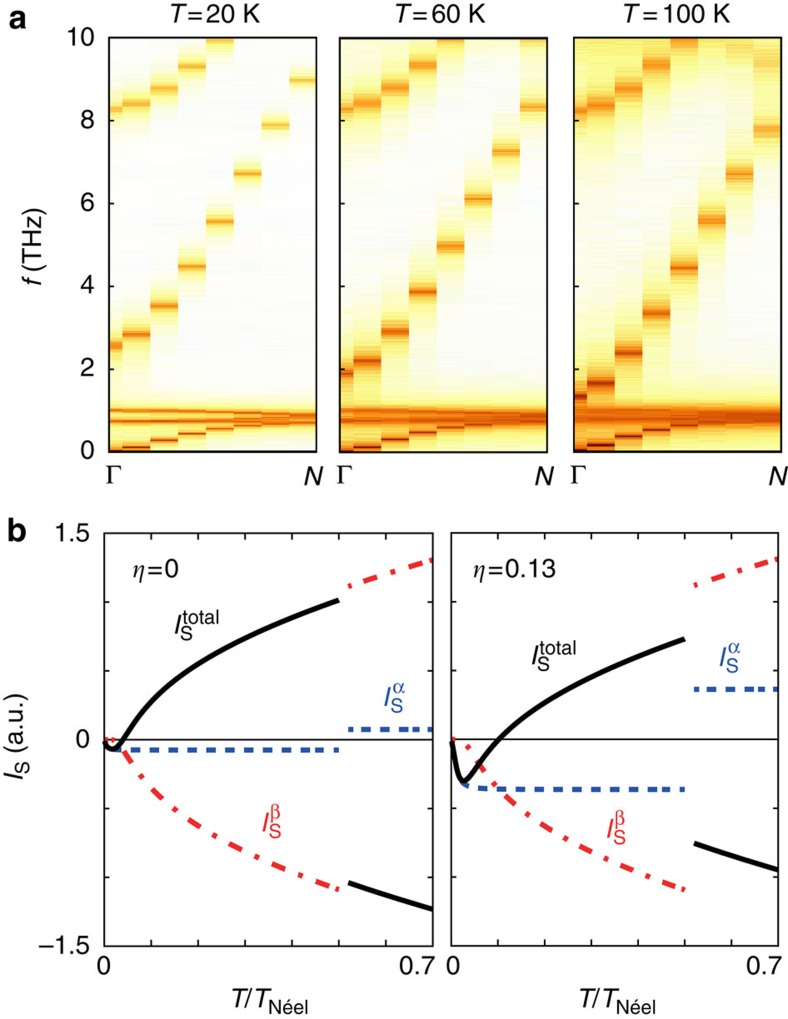
Calculated spin wave spectra of GdIG and sublattice spin currents injected by the SSE. (**a**) Low-frequency spin wave spectra of GdIG calculated from an atomistic model for three different temperatures. The colour code indicates the power spectral density. For temperatures lower than 300 K, the SSE is dominated by two modes: a uniform precession mode in the GHz regime (*α*-mode) and a gapped, optical mode (*β*-mode). The two nearly dispersionless modes around 1 THz are the Gd moments precessing in the exchange field of the Fe moments. (**b**) Temperature dependence of the spin current 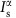 (red) and 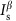 (blue) caused by the respective magnon modes. The total spin current 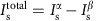 (black) determines the SSE. 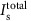 takes the different interface exchange couplings at the GdIG/Pt interface into account. The ratio between these couplings is given by *η*.

First, we discuss the atomistic spin model, which is described in detail in the Supplementary Note 1. The approach is based on the classical Heisenberg model and the Landau–Lifshitz–Gilbert equation, which describes the dynamical behaviour. By including a Langevin thermostat, numerical calculations using the parameters summarized in Supplementary Table 1 yield the spin dynamics including statistical thermal properties (see Supplementary Figures 1 and 2). From the space–time Fourier transform of the real time dynamics, the spin wave spectrum is obtained. Since the model does not involve a magnon ansatz, it includes all non-linear effects such as the magnon–magnon interactions, which are important for the understanding of the relative contributions of each sublattice to the SSE. By analysing the spin wave spectrum we gain insight into the temperature dependence of the frequency, amplitude, as well as the linewidth of the modes, which goes beyond mean-field theories and the random phase approximation. The thus obtained low-frequency spin wave spectra of GdIG are shown in Fig. 4a. The first band in the GHz regime corresponds to the ferrimagnetic resonance mode (*α*-mode), followed by two nearly dispersionless modes at 1 THz. These two optical modes are the Gd moments precessing in the exchange field of the Fe moments^16^. The next approximately parabolic band is a gapped, optical mode (*β*-mode) precessing in opposite sense as compared with the *α*-mode, where the gap at *k*=0 depends on the Fe–Gd exchange couplings. The higher frequency spin wave modes have only small amplitudes below room temperature (see Supplementary Figure 3) and are disregarded in the further discussion. At low temperature, the *α*-mode dominates the SSE signal. With increasing temperature the two Gd-modes close to 1 THz decrease in power spectral intensity and broaden (see Supplementary Figure 4), reducing their contribution to the SSE due to the decrease in coherence of the associated spin waves. Significantly, the optical *β*-mode red shifts due to the loss of order of the Gd moments, reducing the magnon gap at *k*=0. At a certain temperature the contribution of the *β*-mode overwhelms that of the *α*-mode resulting in a sign change of the SSE due to the different polarization of the *α*-mode compared with the *β*-mode. Therefore, the temperature dependence of the SSE is determined predominantly by just two magnon bands and the shrinking of the magnon gap at *k*=0.

The atomistic model does not address possible interface effects such as a temperature-dependent exchange coupling at the GdIG/Pt interface. Since the measured SSE signal is based on the spin currents injected from the ferrimagnet GdIG into the normal metal Pt, magnon-mode-dependent interface exchange couplings can be important. We carried out theoretical calculations of the SSE using a linear response formalism based on a two spin-lattice model with parametrized interface exchange couplings. As detailed in the Supplementary Note 2, the linear response theory results in two non-degenerate magnon modes^5^: the *α*-mode exhibits a gapless dispersion with a narrow bandwidth, while the magnon spectrum of the other mode (*β*-mode) is gapped by the exchange coupling energy between the Gd and the Fe sublattices. Thus, the two sublattice model is an effective model employed to understand the physics at play. The resulting temperature-dependent spin currents of both modes are shown in Fig. 4b. The total SSE signal in the GdIG/Pt hybrid is given by 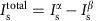. We make the assumption that the interface exchange coupling of the Gd 4*f*-spin with the Pt conduction electrons is weaker than that of the Fe 3*d*-spin. The ratio between these interface couplings is given by *η*. Figure 4b reveals that the temperature of the SSE sign change *T*_sign2_ increases with increasing *η*. Using *η*=0.13 the experimentally obtained temperature *T*_sign2_≈68 K can be reproduced.

## Discussion

We experimentally and theoretically investigated the temperature dependence of the longitudinal SSE in GdIG/Pt bilayers grown on different substrates and with different layer thicknesses. Using two complementary experimental approaches, we find two sign changes of the SSE signal as a function of temperature. The first sign change at *T*_sign1_ close to the magnetic compensation temperature *T*_comp_ can be understood in terms of the reversal of the sublattice magnetizations at *T*_comp_, which preserves the net magnetization along the external magnetic field (Fig. 1). Since the spin current polarization is determined by the (sublattice) magnetization orientation, this leads to a sign change in the SSE current (Fig. 4b), as predicted by Ohnuma *et al*.^5^. The second sign change at *T*_sign2_ can arise from the different temperature dependences of the spin wave power spectral densities and/or different exchange coupling strengths of these modes at the GdIG/Pt interface. Spin wave spectra based on a classical atomistic spin model as a function of temperature reveal that the temperature dependence of the SSE in GdIG is determined predominantly by two modes: a soft-mode with a gapless dispersion and narrow bandwidth (*α*-mode) as well as a gapped, optical mode (*β*-mode), where the gap at *k*=0 depends on the Fe–Gd exchange coupling. Due to the reduced order of the Gd moments, the magnon gap closes with increasing temperature. This causes a sign inversion of the SSE signal at a certain temperature, above which the contribution of the *β*-mode to the SSE becomes larger than that of the *α*-mode. However, the temperature *T*_sign2_ of this SSE sign change might be influenced by different exchange couplings at the GdIG/Pt interface, that is, by different relative weights of the *α*- and *β*-mode spin current transfer into the Pt layer. By assuming a weaker exchange coupling of the *α*-magnons compared with the *β*-magnons at the interface 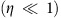, not only the magnitude of *T*_sign2_ but also the gradual change of the SSE signal as a function of temperature can be reproduced (Fig. 4b). Concluding, the origin of the second SSE sign change results from an increased contribution of the gapped *β*-mode with increasing temperature. In addition, the temperature at which the second sign change occurs might be affected by mode-dependent exchange coupling strengths at the GdIG/Pt interface. Thus, the SSE probes the complex spin wave dynamics in ferrimagnets.

## Methods

### Sample fabrication

Three different sets of samples were fabricated and investigated: at Mainz, GdIG films were deposited on single crystalline, (100)-oriented gadolinium gallium garnet (Gd_3_Ga_5_O_12_, GGG) substrates by pulsed laser deposition using a KrF excimer laser with a laser fluence of 1.2 J cm^−2^. We here focus on a 100-nm-thick GdIG film (sample A) grown with optimized parameters, that is, in an oxygen atmosphere at 2 × 10^−2^ mbar and at a substrate temperature of 650 °C. No parasitic phases were detected using x-ray diffraction. Furthermore, an out-of-plane lattice constant of (1.254±0.005) nm, which is comparable to the bulk value of 1.247 nm, is calculated using the GdIG (400) film reflection. Moreover, the rocking curve around the GdIG (400) reflection has a full width at half maximum (FWHM) of 0.036°, indicating a high crystalline quality and low mosaicity. The 8 nm-thick Pt layer, used for the ISHE measurements, was deposited by DC-magnetron sputtering after *in situ* cleaning of the interface. At Sendai, a GdIG/Pt bilayer was fabricated by liquid phase epitaxy of GdIG with a thickness of around 1 μm on a (111)-oriented GGG substrate and subsequently depositing a 10 nm-thick Pt layer by DC-magnetron sputtering on the GdIG thin film (sample B). The FWHM of the rocking curve around the GdIG (444) reflection is comparable to the FWHM value of the rocking curve around the GGG (444) substrate reflection, demonstrating the high crystalline quality of the GdIG thin film. At Garching, the GdIG films were deposited onto single crystalline, (111)-oriented yttrium aluminium garnet (Y_3_Al_5_O_12_, YAG) substrates via pulsed laser deposition. Best structural and magnetic properties of the GdIG films were obtained by using a substrate temperature of 500 °C, an oxygen atmosphere of 1.25 × 10^−2^ mbar and an energy fluence of the KrF excimer laser of 2.0 J cm^−2^ at the target surface. The crystalline quality of the GdIG films demonstrated by the FWHM of the rocking curves around the GdIG (444) reflection of around 0.04° is comparable to the GdIG thin films fabricated at Mainz and at Sendai. We here discuss a 26 nm-thick GdIG film with a lattice constant of (1.252±0.005) nm, covered *in situ*, without breaking the vacuum, with 4 nm of Pt deposited via electron beam evaporation (sample C).

### Longitudinal SSE experiments

The longitudinal SSE was recorded as a function of temperature, using two complementary methods. At Mainz and at Sendai, the sample is sandwiched between two heat reservoirs (copper blocks and AlN ceramics, respectively). A resistive heater within each block allows to independently set and stabilize its temperature. In particular, it thus is possible to invert the temperature difference between the heat baths, allowing to exclude spurious effects due to asymmetric sample mounting. In case of the copper blocks, thin single crystalline sapphire plates combined with thermo-conductive foil ensure good thermal contact as well as electric insulation between the copper blocks and the sample. Here the transverse voltage *V*_t_ perpendicular to an external magnetic field is measured as a function of the external magnetic field magnitude at a fixed magnetic field orientation 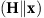. In Garching, the GdIG/Pt bilayer is micropatterned into a Hall bar structure using optical lithography and Argon ion beam milling. The sample is then inserted into a three-dimensional vector magnet cryostat, in which He exchange gas in a variable temperature insert allows us to adjust the sample base temperature. We generate the temperature gradient across the GdIG/Pt interface by sourcing a large current *I*_d_ along the Pt microstructure itself, and exploit the temperature-dependent resistance of the Pt for on-chip thermometry. Here *V*_t_ is recorded as a function of the in-plane external magnetic field direction *α* using a fixed magnetic field magnitude.

## Additional information

**How to cite this article:** Geprägs, S. *et al*. Origin of the spin Seebeck effect in compensated ferrimagnets. *Nat. Commun.* 7:10452 doi: 10.1038/ncomms10452 (2016).

## Supplementary Material

Supplementary Information Supplementary Figures 1-4, Supplementary Table 1, Supplementary Notes 1-2 and Supplementary References
