# Supplementary material for: Origin of the spin Seebeck effect in compensated ferrimagnets
Source: Nat Commun. 2016 Feb 4;7:10452. doi: 10.1038/ncomms10452 (PMC4742853; doi:10.1038/ncomms10452)
Supplement: Supplementary Information — Supplementary Figures 1-4, Supplementary Table 1, Supplementary Notes 1-2 and Supplementary References [file ncomms10452-s1.pdf]

Supplementary Note 1

In this section we present the spin wave power spectra of Gadolinium Iron Garnet ($Gd_3Fe_5O_{12}$) as calculated numerically in a classical atomistic spin model. They are a direct measure of thermal spin pumping and the resulting spin Seebeck (SSE) voltage: At low temperature, the SSE is dominated by the uniform ferrimagnetic resonance mode and takes the conventional sign. With increasing temperature the exchange splitting between Fe and Gd is reduced leading to an enhanced contribution from the gapped, optical mode to the SSE. At a certain temperature this contribution becomes even larger than that of the uniform ferrimagnetic resonance mode causing a sign reversal of the SSE. At the magnetization compensation temperature the Fe sublattices dominate and the change in their orientations leads to the second sign change.

We first consider the classical Heisenberg model

$$\mathcal{H} = -\sum_{\langle ij \rangle} J_{ij} \mathbf{S}_i \cdot \mathbf{S}_j - \sum_i d_z S_{z,i} \tag{1}$$

where J_{ij} is the exchange energy between two classical spin vectors $\mathbf{S}_i$ and $\mathbf{S}_j$ ($|\mathbf{S}| = 1$), d_z is an uniaxial energy with easy axis along the z-direction that defines the quantization axis for the spin waves. We model the entire GdIG unit cell of 32 magnetic moments using exchange couplings derived from inelastic neutron diffraction experiments (Supplementary Table 1) [3].

symbol	value	units	description	source
$S(Fe^{3+})$	5/2		spin	[1]
$S(Gd^{3+})$	7/2			
μ_s(Fe)	5.92	μ_B	magnetic moment	$g\sqrt{S(S+1)}$
μ_s(Gd)	7.95	μ_B		
α_Fe	0.002		Gilbert damping parameter	[2]
α_Gd	0.006			
γ	1.00	γ_e	gyromagnetic ratio	
J_{aa}	-1.10×10^{-21}	J/bond	exchange energy	[3]
J_{dd}	-1.10×10^{-21}			
J_{ad}	-5.52×10^{-21}			
J_{ac}	-4.08×10^{-22}			
J_{dc}	-1.64×10^{-21}			

Supplementary Table 1: Parameters used in the numerical model.

The spin dynamics is described by the Landau-Lifshitz-Gilbert equation for spin $\mathbf{S}_i$ with local magnetic field $\mathbf{H}_i$ and damping parameter α_i

$$\frac{\partial \mathbf{S}_i}{\partial t} = -\frac{\gamma_i}{(1+\alpha_i^2)} \left(\mathbf{S}_i \times \mathbf{H}_i + \alpha_i \mathbf{S}_i \times (\mathbf{S}_i \times \mathbf{H}_i) \right) \tag{2}$$

From the solutions the magnetization dynamics of sublattice n is calculated as

$$M_n(t) = \frac{1}{N_n} \sum_{i \in n} \mathbf{S}_{i,n}(t) \quad . \tag{3}$$

The Gilbert damping parameters for the Fe ($S = 5/2$) and Gd ($S = 7/2$) moments have been measured in GdIG where α_Gd was extracted from the FMR linewidth under the assumption that $\alpha_\mathrm{Fe}(\mathrm{GdIG}) = \alpha_\mathrm{Fe}(\mathrm{YIG})$ [2]. We do not include any temperature dependence of the exchange constants and intrinsic damping parameters, thereby assuming that the experimental findings are dominated by the thermal fluctuations. This simplification allows us to deconvolute the contribution of magnon-magnon interactions to the damping from changes due to spin-lattice coupling as encapsulated by the Gilbert and exchange parameters.

The effective local magnetic field on a lattice site i is

$$\mathbf{H}_i = -\frac{1}{\mu_s} \frac{\partial \mathcal{H}}{\partial \mathbf{S}_i} + \boldsymbol{\xi}_i \tag{4}$$

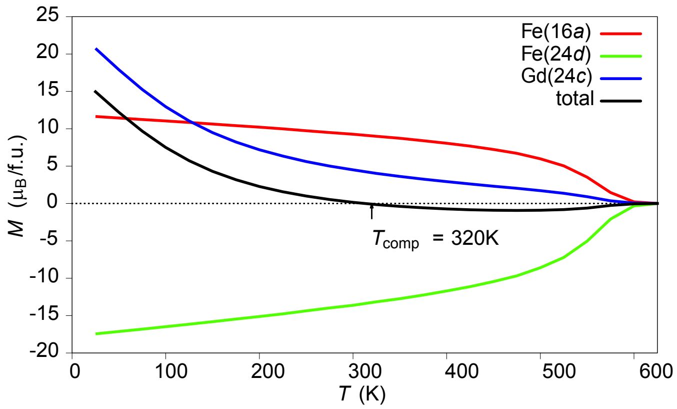

Supplementary Figure 1: Temperature dependence of the magnetization of each sublattice and the total.

where $\mu_s = g\sqrt{S(S+1)}\mu_B$ ($g \approx 2$ is the g-factor) is the magnetic moment. Since $\boldsymbol{\xi}_i$ is a field-like stochastic process, Supplementary Equation (2) is a Langevin equation [4]. Here we consider the white noise limit with correlators

$$\langle \boldsymbol{\xi}_i(t) \rangle = 0; \quad \langle \boldsymbol{\xi}_{i,a}(t), \boldsymbol{\xi}_{j,b}(t') \rangle = (2k_B T \alpha_i / \mu_i \gamma_i)\delta(|t-t'|)\delta_{ij}\delta_{ab} \tag{5}$$

where a and b are Cartesian components. The white noise approximation is valid as long as the eigenfrequencies $\omega \ll k_B T/\hbar$. Since we are concerned with frequencies of the order 2 THz and below, this holds even down to 20 K.

The spin Seebeck effect is caused by the temperature gradient-induced imbalance between the thermally induced spin pumping and the spin transfer torque exerted by the Johnson Nyquist noise in the normal metal [5]. Both contributions are proportional to the presumably temperature-independent spin mixing conductance as well as integrals over the dynamic magnetic susceptibility at the interface. The latter are in turn integrals over wave vector and momentum of the dynamic structure factor (DSF)

$$\mathcal{S}(\mathbf{q},\omega) = \frac{1}{\mathcal{N}\sqrt{2\pi}} \sum_{\mathbf{r},\mathbf{r}'} \mathrm{e}^{\mathrm{i}\mathbf{q}\cdot(\mathbf{r}-\mathbf{r}')} \int_{-\infty}^{+\infty} \mathrm{e}^{\mathrm{i}\omega t}\, C(\mathbf{r}-\mathbf{r}',t)\,\mathrm{d}t \tag{6}$$

where

$$C(\mathbf{r}-\mathbf{r}',t) = \langle S_+(\mathbf{r},0) S_-(\mathbf{r}',t) \rangle \tag{7}$$

is the space-time spin-spin correlation function of the transverse components (x,y). Structures in the DSF reflect the energy dispersion and the thermal broadening of the spin waves, where the latter is induced by the magnon-magnon interactions. The thermal spin pumping contribution to the spin Seebeck effect is governed by an integral over frequency and momentum of the DSF (weighted by frequency) [5]. Here we approximate the interface DSF by that of the bulk ferromagnet and focus on the apparent qualitative physics that governs the sign change of the SSE such as the relative contributions of each sublattice.

Our numerical method achieves a good ensemble average and sampling of the Brillouin zone for a system of $16 \times 16 \times 16$ unit cells ($\mathcal{N} = 262144$) with periodic boundary conditions. The system is equilibrated via a two stage process: First, an over-damped Langevin simulation rapidly brings the system to thermal equilibrium, while a subsequent longer equilibration with the damping parameters in the Supplementary Table 1 prepares the initial conditions. The LLG equation is then solved for 500 ps (time step $\Delta t = 0.1$ fs) generating the DSF with a high resolution even at low frequencies. The present parameter set predicts a Curie temperature $T_C = 600$ K and a magnetization compensation temperature $T_{comp} = 320$ K both approximately 50 K higher than the experimental values (Supplementary Figure 1). This temperature offset does not change the interpretation of our results in the context of the experiments as the phenomena we describe belong to the class of ferrimagnets with a weakly coupled sublattice [6].

The SSE is proportional to the transverse magnetization dynamics. Supplementary Figure 2 shows the real-time magnetization dynamics, which emphasizes the weak temperature dependence of the transverse magnetization of the Gd relative to that of the Fe moments. Hence, the contribution of the Gd moments to the SSE decreases only slightly as a function of temperature. This trend can be explained by the spin wave spectra as calculated from the DSF in Supplementary Figure 3 that provide the temperature dependence of the frequency, amplitude and linewidths. Using

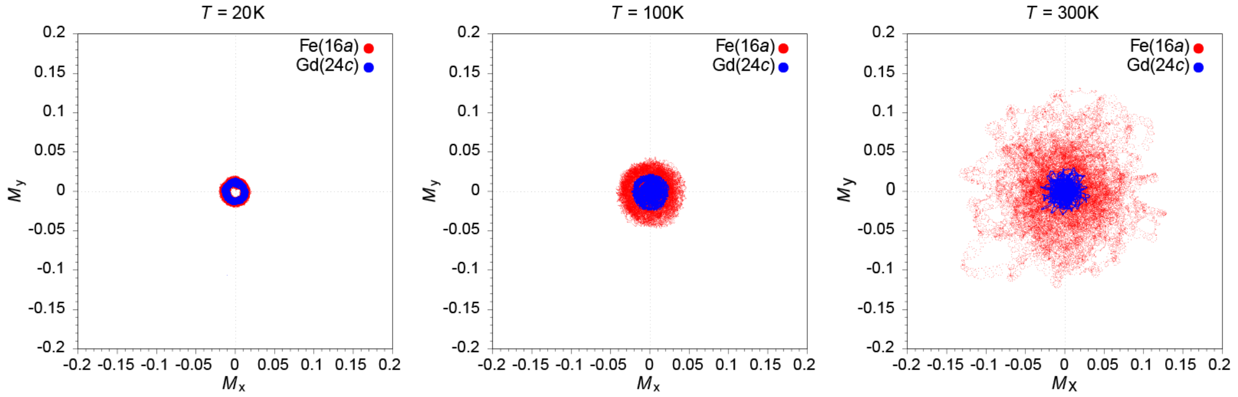

Supplementary Figure 2: Comparison of the Fe (red dots) and Gd (blue dots) transverse magnetization dynamics for different temperatures.

a numerical approach takes us beyond mean-field theories with a more complete description of the thermodynamics and the inclusion of magnon-magnon interactions.

The low temperature spin wave spectra in Supplementary Figures 3a-c agree well with analytic approximations [3]. As already discussed in the main text, the lowest frequency peak, in the GHz regime, corresponds to the ferrimagnetic resonance (FMR). It dominates the spectrum with a large oscillator strength and small broadening that reflects the weak intrinsic damping of GdIG. The two flat bands with frequencies close to 1 THz are attributed to the Gd moments precessing in the exchange field from the Fe moments [3]. The next approximately parabolic band is caused by the spin splitting of the rare-earth ion where the gap at $k = 0$ depends on the Fe-Gd exchange couplings as $-10J_{dc} + 20J_{ac}$ [3].

At low temperature the spectrum is dominated by the FMR mode and the higher frequency dynamics are mostly frozen out by the large magnon gap. Hence the SSE takes the conventional sign where the Gd and Fe a sublattice determines the chirality of the precession.

With increasing temperature, the Gd central peaks close to 1 THz decrease in power spectral intensity and broaden, reflecting a decrease in coherence of the associated spin waves governed by the weak Fe-Gd exchange coupling. However, this effect is small compared to the significant increase of the power spectral density of the optical mode with parabolic dispersion at low k-values, which red shifts with increasing temperature. This point is emphasized more clearly by the (smoothed) frequency scans at $q = 0$ shown for different temperatures in Supplementary Figure 4. The integration of features in the DSF give their contribution to the SSE. The large line width and increase in power spectral density of the red-shifting mode with increasing temperature leads to an increasing contribution to the SSE with opposite polarization to the FMR mode. The band edge of this mode is determined by the spin splitting of the Gd. The gap is highly temperature dependent because the Gd rapidly loses order due to the weak Fe-Gd exchange coupling. This is seen in Supplementary Figure 1 and by the broadening of the flat Gd bands in the DSF. At some point the SSE contribution of the gapped mode becomes even larger than that of the FMR mode (and other modes of the same polarization). Then the sign of the SSE becomes opposite to the conventional sign. The precise temperature where this occurs is difficult to determine as it requires the integration of the DSF over the Brillouin zone. The quantitative analysis is beyond the scope of this work and is reserved for a future challenging study. However, we can draw the important qualitative conclusion that the temperature dependence of the SSE is determined predominantly by just two magnon bands and the closing of the magnon gap.

The high-temperature change of the sign of the SSE at the magnetization compensation point is caused by the reorientation of the sublattice magnetizations as predicted in Ref. 7. When crossing the compensation temperature the two branches exchange roles, leading to a sign change of the net magnetization and spin pumping. The spin wave spectra shown in Supplementary Figures 3(d-f) around the compensation temperature as well as the corresponding frequency scans at $q = 0$ (Supplementary Figure 4b) clearly show that the Gd modes are weak and hence their contribution is negligibly small. Moreover, the spin splitting of the Gd ions almost vanishes. The FMR and optical modes are also almost degenerate.

Most of the materials used in SSE experiments are ferrimagnets with multiple magnetic sublattices and complex spin wave spectra that cannot be fully understood by simple analytical models. The results and insight gained through these simulations demonstrate the contributions of multiple spin wave bands to the SSE.

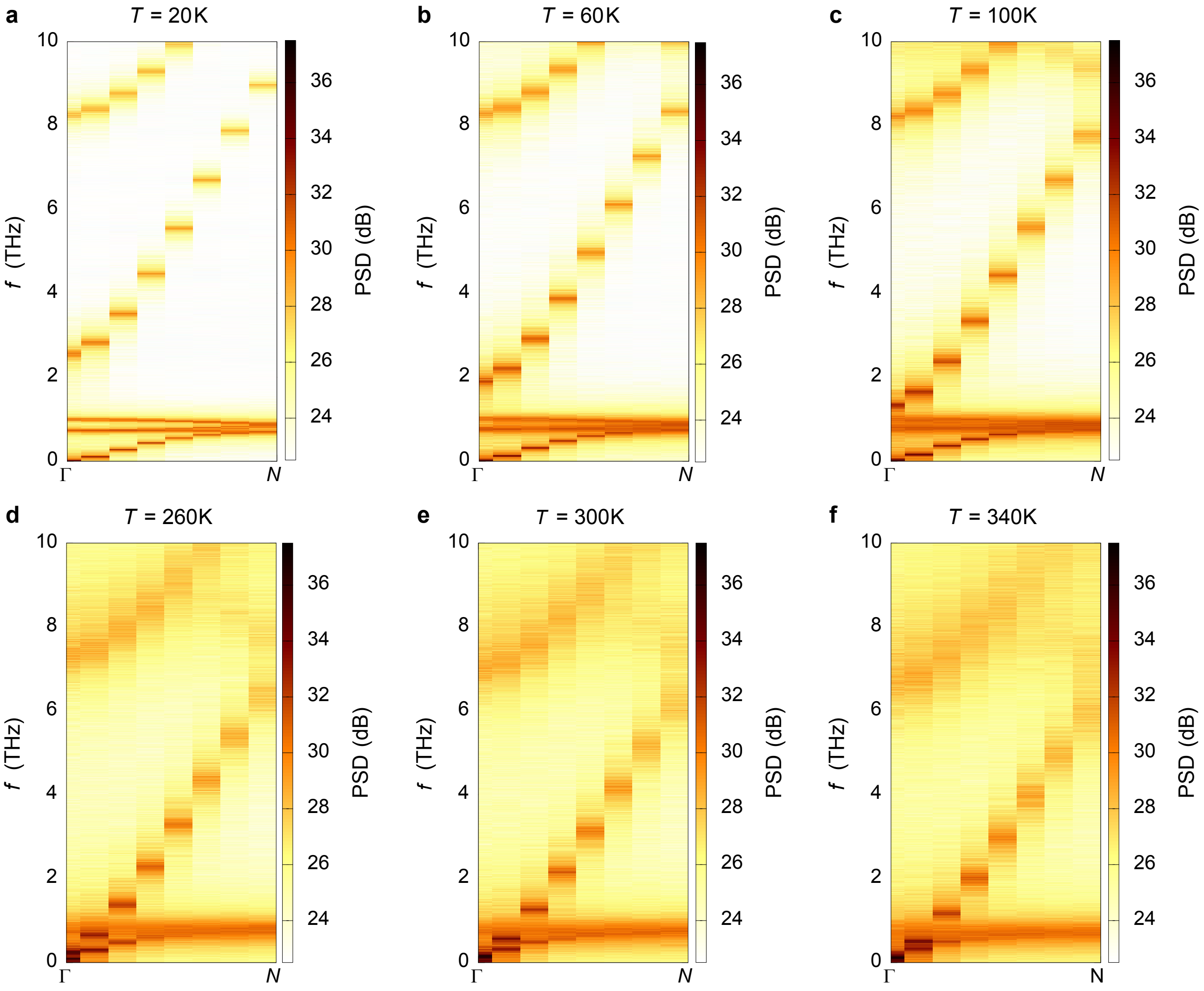

Supplementary Figure 3: Low-frequency spin wave spectra of GdIG calculated from finite temperature spin dynamics simulations. The color code indicates the power spectral density (PSD): (a-c) The spin wave spectra at low temperatures up to the Gd ordering temperature, (d-f) around the magnetization compensation temperature of $T_{\mathrm{comp}} = 320\,\mathrm{K}$.

Supplementary Note 2

In this section, we outline a linear response approach to the spin Seebeck effect (SSE) in a compensated ferrimagnet [7]. We demonstrate that the changes of sign of the SSE observed in ferrimagnet insulator (FMI, in the experiment $Gd_3Fe_5O_{12}$)/paramagnetic metal (NM, in the experiment Pt) hybrid structures can be accounted for by a model calculation within the linear response formalism that uses realistic values of the interface (s-d or s-f) exchange coupling. We shall show below that the first sign change of the SSE at a higher temperature T_{sign1} originates from the reversal of the spin quantization axis at the magnetization compensation temperature T_{comp}, whereas the second sign change at a lower temperature T_{sign2} is caused by a competition between a soft-mode magnon and a gapped-mode magnon. The former mode, which corresponds to the FMR mode in a first order treatment, is mainly composed of Gd spins and has a weak exchange coupling at the FMI/NM interface. The latter mode, which corresponds to an optical mode, is mainly composed of Fe spins and has a stronger exchange coupling at the FMI/NM interface than the FMR mode.

Let us begin with a brief review of a linear response theory for the SSE in a compensated ferrimagnet [7, 8]. Our starting point is the following model Hamiltonian for a compensated ferrimagnet FMI with two different spins $\mathbf{S}_{\mathrm{A}}$

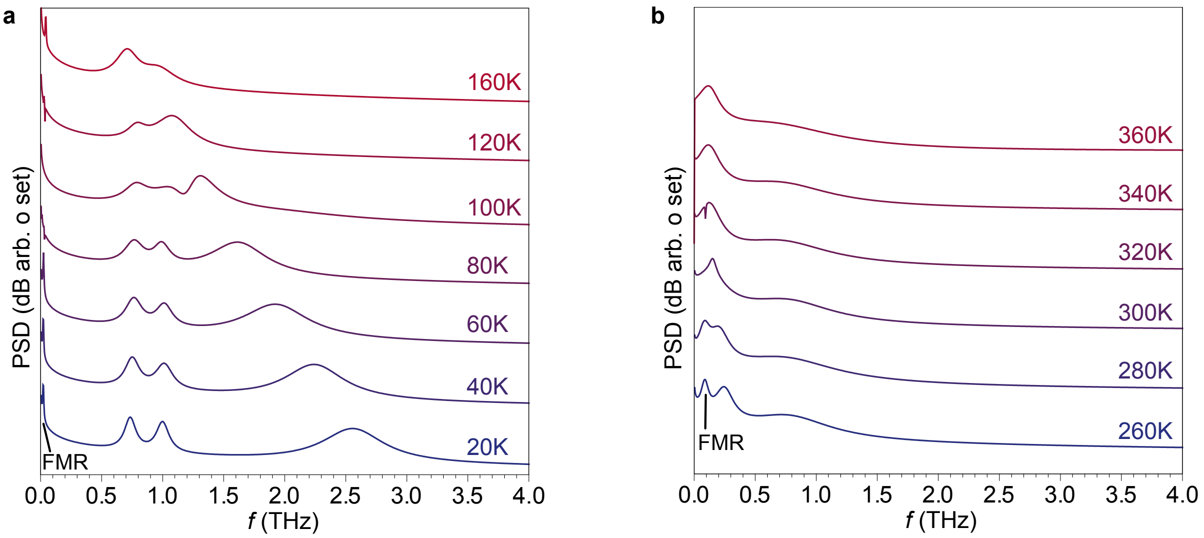

Supplementary Figure 4: Frequency scans of the spin wave power spectral density (PSD) at $\mathbf{q} = 0$ for various temperatures. (a) around the Gd ordering temperature, (b) around the magnetization compensation temperature $T_{\rm comp}$.

and $\mathbf{S}_{\rm B}$ defined on two sublattices A and B [7]:

$$\mathcal{H}_{\rm FMI} = -J_{\rm A} \sum_{\langle i,i' \rangle \in {\rm A}} \mathbf{S}_{{\rm A},i} \cdot \mathbf{S}_{{\rm A},i'} - J_{\rm B} \sum_{\langle j,j' \rangle \in {\rm B}} \mathbf{S}_{{\rm B},j} \cdot \mathbf{S}_{{\rm B},j'} + J_{AB} \sum_{\langle i \in {\rm A}, j \in {\rm B} \rangle} \mathbf{S}_{{\rm A},i} \cdot \mathbf{S}_{{\rm B},j}$$
$$+ \sum_{i \in {\rm A}} \left[g_{\rm A} \mu_{\rm B} \mathbf{H}_0 \cdot \mathbf{S}_{{\rm A},i} - \frac{D_{\rm A}}{2} (\hat{\mathbf{z}} \cdot \mathbf{S}_{{\rm A},i})^2 \right] + \sum_{i \in {\rm B}} \left[g_{\rm B} \mu_{\rm B} \mathbf{H}_0 \cdot \mathbf{S}_{{\rm B},i} - \frac{D_{\rm B}}{2} (\hat{\mathbf{z}} \cdot \mathbf{S}_{{\rm B},i})^2 \right] , \quad (8)$$

where $J_{\rm A}$ and $J_{\rm B}$ (J_{AB}) are the nearest-neighbor intra-sublattice (inter-sublattice) exchange integrals, $\langle i, i' \rangle$ specifies nearest neighbor bonding, $\mu_{\rm B}$ is the Bohr magneton, $\mathbf{H}_0 = -H_0 \hat{\mathbf{z}}$ is the external magnetic field, $g_{\rm L}$ (L = A, B) is the effective g factor, and $D_{\rm L}$ is the anisotropy constant. In this model, a Gd spin with a larger size $S_{\rm A}$ is represented by $\mathbf{S}_{\rm A}$ on sublattice A, whereas an Fe spin with a smaller size $S_{\rm B}$ is represented by $\mathbf{S}_{\rm B}$ on sublattice B.

Using the spin-wave approximation, Supplementary Equation (8) is diagonalized to be [7]

$$\mathcal{H}_{\rm FMI} = \hbar \sum_{\mathbf{q}} \left(\omega^{\alpha}_{\mathbf{q}} \alpha^{\dagger}_{\mathbf{q}} \alpha_{\mathbf{q}} + \omega^{\beta}_{\mathbf{q}} \beta^{\dagger}_{\mathbf{q}} \beta_{\mathbf{q}} \right) , \quad (9)$$

where $\omega^{\alpha}_{\mathbf{q}} = \frac{1}{2} \left[\sqrt{(\epsilon^{\rm A}_{\mathbf{q}} + \epsilon^{\rm B}_{\mathbf{q}})^2 - 4\zeta^2_{\mathbf{q}}} + (\epsilon^{\rm A}_{\mathbf{q}} - \epsilon^{\rm B}_{\mathbf{q}}) \right]$, $\omega^{\beta}_{\mathbf{q}} = \frac{1}{2} \left[\sqrt{(\epsilon^{\rm A}_{\mathbf{q}} + \epsilon^{\rm B}_{\mathbf{q}})^2 - 4\zeta^2_{\mathbf{q}}} - (\epsilon^{\rm A}_{\mathbf{q}} - \epsilon^{\rm B}_{\mathbf{q}}) \right]$, $\epsilon^{\rm A}_{\mathbf{q}} = 2z_0 J_{\rm A} S_{\rm A} [1 - \gamma_{\mathbf{q}}] + z_0 J_{AB} S_{\rm B} + (g_{\rm A} \mu_{\rm B} H_0 + D_{\rm A} S_{\rm A})$, $\epsilon^{\rm B}_{\mathbf{q}} = 2z_0 J_{\rm B} S_{\rm B} [1 - \gamma_{\mathbf{q}}] + z_0 J_{AB} S_{\rm A} + (-g_{\rm B} \mu_{\rm B} H_0 + D_{\rm B} S_{\rm B})$. Here, $\gamma_{\mathbf{q}} = z_0^{-1} \sum_{\boldsymbol{\delta}} e^{{\rm i}\mathbf{q} \cdot \boldsymbol{\delta}}$ is defined by the sum over z_0 nearest neighbors of the original lattice whereas $\zeta_{\mathbf{q}} = J_{AB} \sqrt{S_{\rm A} S_{\rm B}} \sum_{\boldsymbol{\delta}'} e^{{\rm i}\mathbf{q} \cdot \boldsymbol{\delta}'}$ is defined by the sublattice A or B. The magnon operators $\alpha_{\mathbf{q}}$ and $\beta_{\mathbf{q}}$ are related to the Holstein-Primakoff operators $a_{\mathbf{q}}$ and $b_{\mathbf{q}}$ by the Bogoliubov transformation

$$a_{\mathbf{q}} = u_{\mathbf{q}} \alpha_{\mathbf{q}} + v_{\mathbf{q}} \beta^{\dagger}_{\mathbf{q}} , \quad (10)$$
$$b_{\mathbf{q}} = v_{\mathbf{q}} \alpha^{\dagger}_{\mathbf{q}} + u_{\mathbf{q}} \beta_{\mathbf{q}} , \quad (11)$$

where $u_{\mathbf{q}} = \sqrt{\frac{1}{2}} \left[\frac{\epsilon^{\rm A}_{\mathbf{q}} + \epsilon^{\rm B}_{\mathbf{q}}}{[(\epsilon^{\rm A}_{\mathbf{q}} + \epsilon^{\rm B}_{\mathbf{q}})^2 - 4\zeta^2_{\mathbf{q}}]^{1/2}} + 1 \right]^{1/2}$ and $v_{\mathbf{q}} = -{\rm sgn}(\zeta_{\mathbf{q}}) \sqrt{\frac{1}{2}} \left[\frac{\epsilon^{\rm A}_{\mathbf{q}} + \epsilon^{\rm B}_{\mathbf{q}}}{[(\epsilon^{\rm A}_{\mathbf{q}} + \epsilon^{\rm B}_{\mathbf{q}})^2 - 4\zeta^2_{\mathbf{q}}]^{1/2}} - 1 \right]^{1/2}$. In the following we set $S_A = 3 \times 7/2$ and $S_B = 5/2$, because there are three Gd 7/2-spins in each formula unit of $Gd_3Fe_5O_{12}$ while three Fe spins on octahedral sites and two Fe spins on tetrahedral sites align in the opposite sense, giving a resultant 5/2-spin per formula unit [9]. Also, we set $D_{\rm A} = D_{\rm B} = 0\,{\rm meV}$ to simplify the argument, and use $J_{\rm A} = 0\,{\rm meV}$, $J_{\rm B} = 0.68\,{\rm meV}$, $J_{AB} = 0.065\,{\rm meV}$, $g_{\rm A} = 2.0$, $g_{\rm B} = 2.0$, in order to reproduce the Néel temperature ($T_{\rm Néel} = 565\,{\rm K}$) and the compensation temperature ($T_{comp} = 286\,{\rm K}$) [10]. These parameters bring about a situation where the α magnon spectrum is gapless and very soft with a quite narrow bandwidth, whereas the β magnon spectrum is gapped by an amount $\Delta = z_0 J_{\rm AB} (S_{\rm A} - S_{\rm B})$ with a much wider bandwidth (see Figure 2 in Ref. [7]).

Thus, the two sublattice model is an effective model employed to understand the physics at play. Moreover, in the present approach, the two Fe spins on octahedral a sites and the three Fe spins on tetrahedral d sites are considered to be collinear forming a macrospin due to the strong antiferromagnetic exchange interaction J_{ad}. This approximation is justified, since the out-of-phase dynamics of Fe(a) and Fe(d) spins, which breaks the collinear alignment of the Fe spins, introduces an additional mode at around 8 THz (Supplementary Figure 3), however, this mode can be safely neglected due to the large energy gap.

The resultant SSE signal in the FMI/NM hybrid structure under a temperature bias ΔT, represented in a form of the injected spin current I_s, is given by the following formula [7]:

$$I_s = I_s^{\alpha} - I_s^{\beta} \ , \tag{12}$$

$$I_s^{\alpha(\beta)} = \frac{N_{\rm int}}{\hbar^2 N_{\rm NM} N_{\rm FMI}} \sum_{\mathbf{k},\mathbf{q}} \langle [J_{\rm sd}^{\alpha(\beta)}(\mathbf{q})]^2 \rangle {\rm Im}\chi^R(\mathbf{k}, \omega_{\mathbf{q}}^{\alpha(\beta)}) \left(-\frac{\partial N_T(\omega_{\mathbf{q}}^{\alpha(\beta)})}{\partial T} \right) \Delta T \ , \tag{13}$$

where $N_{\rm FMI}$ ($N_{\rm NM}$) is the number of lattice sites in FMI (NM), $N_{\rm int}$ is the number of localized spins per sublattice at the FMI/NM interface, $N_T(\omega) = (e^{\hbar\omega/k_{\rm B}T} - 1)^{-1}$ is the Bose distribution function, and $\chi^R(\mathbf{k}, \omega) = \chi_{\rm NM}/(1 + \lambda^2 k^2 - {\rm i}\omega\tau_{\rm sf})$ is the retarded part of the dynamical spin susceptibility of NM with $\chi_{\rm NM}$, λ and $\tau_{\rm sf}$ being the uniform susceptibility, the spin diffusion length, and the spin-flip relaxation time. In Supplementary Equation (13), the following two factors

$$\langle [J_{\rm sd}^{\alpha}(\mathbf{q})]^2 \rangle = \left[S_{\rm A}(J_{\rm sd}^{\rm A})^2 + S_{\rm B}(J_{\rm sd}^{\rm B})^2 \right] v_{\mathbf{q}}^2 + S_{\rm A}(J_{\rm sd}^{\rm A})^2 \ , \tag{14}$$

$$\langle [J_{\rm sd}^{\beta}(\mathbf{q})]^2 \rangle = \left[S_{\rm A}(J_{\rm sd}^{\rm A})^2 + S_{\rm B}(J_{\rm sd}^{\rm B})^2 \right] v_{\mathbf{q}}^2 + S_{\rm B}(J_{\rm sd}^{\rm B})^2 \ , \tag{15}$$

represent effective interface exchange couplings felt by α- and β-magnons, respectively, and they become important for a later discussion on the second sign change of the SSE that occurs at a lower temperature $T_{\rm sign2}$. In Supplementary Equations. (14) and (15), $J_{\rm sd}^{\rm A}$ and $J_{\rm sd}^{\rm B}$ are defined by the following exchange interaction at the FMI/NM interface:

$$\mathcal{H}_{\rm sd} = \sum_{i,j \in {\rm interface}} \left(J_{\rm sd}^{\rm A} \boldsymbol{\sigma}_i \cdot \mathbf{S}_{{\rm A},i} + J_{\rm sd}^{\rm B} \boldsymbol{\sigma}_j \cdot \mathbf{S}_{{\rm B},j} \right) \ , \tag{16}$$

where $\boldsymbol{\sigma}_i$ is the conduction-electron spin density in NM.

Taking different exchange couplings at the GdIG/Pt into account, we define

$$\eta \equiv J_{\rm sd}^{\rm A} / J_{\rm sd}^{\rm B} \ . \tag{17}$$

In Ref. [7], $\eta = 1$ was assumed throughout the calculation, which predicted only one sign change of the SSE at the magnetization compensation temperature T_{comp}. However, since the exchange interaction of the Gd $4f$-spin with conduction-electron spin is much weaker than that of Fe $3d$-spin, $J_{\rm sd}^{\rm A}$ coming from a Gd spin is much smaller than $J_{\rm sd}^{\rm B}$ coming from an Fe spin in Supplementary Equation (16), giving $\eta \ll 1$. In Figure 4b in the main text, we plot the temperature dependence of the SSE signal by setting $\eta = 0$. Although we can see two sign changes in the SSE signal with the use of this parameter, the calculated value of $T_{\rm sign2} \approx 30$ K is slightly lower than the experimentally observed value of $T_{\rm sign2} \approx 68$ K. The calculated value of $T_{\rm sign2}$ increases upon increasing the η value from zero, and when we use $\eta = 0.13$ (Figure 4b in the main text), we obtain $T_{\rm sign2} \approx 68$ K, which agrees with the experimentally observed value. Thus, the present calculation shows that the two sign changes of the SSE signal observed in $Gd_3Fe_5O_{12}$/Pt hybrid structures can be accounted for by the linear response approach. Moreover, the present calculation demonstrates that we can determine the relative strength of the two interface exchange couplings, i.e., the parameter η defined by Supplementary Equation (17), by measuring $T_{\rm sign2}$ in the SSE signal.

The physics behind the two sign changes is as follows. The origin of the first sign change at $T_{\rm sign1}$ that was already predicted in Ref. [7] is the reversal of the spin quantization axis at the magnetization compensation point $T_{\rm comp}$ when the magnetization direction is pinned by an external magnetic field. On the other hand, the second sign change at $T_{\rm sign2}$ is given by the following two factors: i) the gap $\Delta = z_0 J_{AB}(S_{\rm A} - S_{\rm B})$ in the β-magnon spectrum, and ii) the relation $\langle [J_{\rm sd}^{\alpha}(\mathbf{q})]^2 \rangle \ll \langle [J_{\rm sd}^{\beta}(\mathbf{q})]^2 \rangle$ in Supplementary Equation (13) under the present condition $J_{\rm sd}^{\rm A} \ll J_{\rm sd}^{\rm B}$ that gives $I_s^{\alpha} \ll I_s^{\beta}$ in the high temperature limit. As obvious from Fig. 4b in the main text, at low temperatures $T \lesssim \Delta/k_{\rm B}$, the SSE signal is dominated by α-magnon because of the gap Δ in the β-magnon spectrum. At higher temperatures $T \gg \Delta/k_{\rm B}$, however, the SSE signal is mainly given by β-magnon because of the relation $\langle [J_{\rm sd}^{\alpha}(\mathbf{q})]^2 \rangle \ll \langle [J_{\rm sd}^{\beta}(\mathbf{q})]^2 \rangle$ in Supplementary Equation (13). Since I_s^{α} and I_s^{β} contribute opposite in sign through Supplementary Equation (12), this competition gives rise to a sign change at a temperature $T_{\rm sign2}$. Note that if we use $J_{\rm sd}^{\rm A} = J_{\rm sd}^{\rm B}$ as was done in Ref. [7], the second sign change does not appear because the α- and β-magnon mode contributions become comparable in the high temperature limit.

Supplementary References

[1] Oitmaa, J. & Falk, T. Ferrimagnetism in the rare-earth iron garnets: a monte carlo study. *J. Phys.: Condens. Matter* **21**, 124212 (2009).

[2] Smith, W. V., Overmeyer, J. & Calhoun, B. A. Microwave resonance in gadolinium-iron garnet crystals. *IBM J. Res. Dev.* **3**, 153–162 (1959).

[3] Harris, A. B. Spin-wave spectra of yttrium and gadolinium iron garnet. *Phys. Rev.* **132**, 2398–2409 (1963).

[4] García-Palacios, J. & Lázaro, F. Langevin-dynamics study of the dynamical properties of small magnetic particles. *Phys. Rev. B* **58**, 14937–14958 (1998).

[5] Xiao, J., Bauer, G. E. W., Uchida, K., Saitoh, E. & Maekawa, S. Theory of magnon-driven spin seebeck effect. *Phys. Rev. B* **81**, 214418 (2010).

[6] Wolf, W. P. Ferrimagnetism. *Rep. Prog. Phys.* **24**, 212–303 (1961).

[7] Ohnuma, Y., Adachi, H., Saitoh, E. & Maekawa, S. Spin seebeck effect in antiferromagnets and compensated ferrimagnets. *Phys. Rev. B* **87**, 014423 (2013).

[8] Adachi, H., Ohe, J.-i., Takahashi, S. & Maekawa, S. Linear-response theory of spin seebeck effect in ferromagnetic insulators. *Phys. Rev. B* **83**, 094410 (2011).

[9] Kittel, C. *Introduction to Solid State Physics* (John Wiley & Sons, New York, 2005), 8th ed. edn.

[10] Chikazumi, S. *Physics of Ferromagnetism* (Oxford University Press, Oxford, 1997), 2nd ed. edn.
